# Utilization of Isoflavones in Soybeans for Women with Menopausal Syndrome: An Overview

**DOI:** 10.3390/ijms22063212

**Published:** 2021-03-22

**Authors:** Li-Ru Chen, Kuo-Hu Chen

**Affiliations:** 1 Department of Physical Medicine and Rehabilitation, Mackay Memorial Hospital, Taipei 10049, Taiwan; gracealex168@gmail.com; 2 Department of Mechanical Engineering, National YangMing ChiaoTung University, Hsinchu 30010, Taiwan; 3 Department of Obstetrics and Gynecology, Taipei Tzu-Chi Hospital, The Buddhist Tzu-Chi Medical Foundation, Taipei 231, Taiwan; 4 School of Medicine, Tzu-Chi University, Hualien 970, Taiwan

**Keywords:** menopause, soybeans, isoflavones

## Abstract

Based on their nutrient composition, soybeans and related foods have been considered to be nutritious and healthy for humans. Particularly, the biological activity and subsequent benefits of soy products may be associated with the presence of isoflavone in soybeans. As an alternative treatment for menopause-related symptoms, isoflavone has gained much popularity for postmenopausal women who have concerns related to undergoing hormone replacement therapy. However, current research has still not reached a consensus on the effects of isoflavone on humans. This overview is a summary of the current literature about the processing of soybeans and isoflavone types (daidzein, genistein, and S-equol) and supplements and their extraction and analysis as well as information about the utilization of isoflavones in soybeans. The processes of preparation (cleaning, drying, crushing and dehulling) and extraction of soybeans are implemented to produce refined soy oil, soy lecithin, free fatty acids, glycerol and soybean meal. The remaining components consist of inorganic constituents (minerals) and the minor components of biologically interesting small molecules. Regarding the preventive effects on diseases or cancers, a higher intake of isoflavones is associated with a moderately lower risk of developing coronary heart disease. It may also reduce the risks of breast and colorectal cancer as well as the incidence of breast cancer recurrence. Consumption of isoflavones or soy foods is associated with reduced risks of endometrial and bladder cancer. Regarding the therapeutic effects on menopausal syndrome or other diseases, isoflavones have been found to alleviate vasomotor syndromes even after considering placebo effects, reduce bone loss in the spine and ameliorate hypertension and in vitro glycemic control. They may also alleviate depressive symptoms during pregnancy. On the other hand, isoflavones have not shown definitive effects regarding improving cognition and urogenital symptoms. Because of lacking standardization in the study designs, such as the ingredients and doses of isoflavones and the durations and outcomes of trials, it currently remains difficult to draw overall conclusions for all aspects of isoflavones. These limitations warrant further investigations of isoflavone use for women’s health.

## 1. Introduction

As a food crop, soybeans have been used in Eastern countries for over 5000 years and serve as an important source of proteins. In recent centuries, soybeans were cultivated and processed for export and became popular in Europe and the United States [1,2,3]. They grow best in tropical, subtropical and temperate regions. Furthermore, they can be grown throughout the year in the tropics and subtropics if water supply is adequate [1,2,3]. The growing time of soybeans ranges from 50 to 200 days, depending on the species, latitude and weather. After soybeans are harvested, threshing consisting of removing the beans from the pods must be done carefully to prevent breakage of the hulls and beans, which can impair the quality [1,2,3]. There are a lot of nonfermented soy foods including soymilk, tofu, soya sprouts, soya film, vegetable or roasted soybeans, and fermented oriental soya foods such as miso, tempeh, soya sauce and natto [1,2,3,4].

Soybeans have an exceptional nutritional and functional food profile. Because soybeans contain practically no starch, they are an important part of a diabetic diet. Soybeans are rich in very high levels of protein, carbohydrate conjugates, fatty acids, soybean oil and amino acids. In addition, soybeans contain several biologically interesting phytochemicals as minor components, consisting of organic and inorganic constituents (minerals). All of the components in soybeans have health impacts on humans, and they can be extracted with different techniques [5,6]. Based on their nutrient composition, soybeans and relative foods have been considered to be nutritious and healthy for humans [1]. There is increasing evidence reporting that consumption of soy foods can significantly affect health [7]. Phytoestrogen-rich soybeans, which are common in Asian diets, are related to a lower incidence of menopausal symptoms, osteoporosis, cardiovascular diseases and hormone-dependent cancers [1]. In addition, there is a significant connection between increased soybean consumption and a decreased cancer risk [4]. Furthermore, a report confirmed that soy proteins could lower blood cholesterol concentrations [4]. Particularly, the biological activity and subsequent benefits of soy products may be associated with the presence of isoflavones in soybeans [3,7]. The US Food and Drug Administration (FDA) approved soy products as an official cholesterol-lowering food along with other heart and health benefits due to the evidence that soy product intake was correlated with significant decreases in serum cholesterol, low-density lipoprotein (LDL) and triglycerides. Nevertheless, it seems to be somewhat difficult to support soy product benefits by in vitro studies using individually isolated phytochemicals (a wide variety of compounds produced by plants) or crude products prepared from soybeans. It is important to note that all phytochemicals isolated from soybeans show an array of weak biological activities; thus, normal consumption of foods that contain these phytochemicals may not provide sufficient amounts to elicit a visible physiological response in humans in short-time clinical research.

Menopause is an aging process that can carry troublesome symptoms including hot flush, sweating and mood changes, and it can also elevate the risk of mortality because of osteoporosis, fractures and decreased physical activity. The most effective way to alleviating menopausal syndrome is hormone replacement therapy (HRT). Nonetheless, a study by the Women’s Health Initiative clearly demonstrated that in postmenopausal women, HRT increased the risks for coronary heart disease, stroke and breast cancer [8]. Since that time, postmenopausal women and healthcare workers have been searching for an alternative therapy. A Canadian study has shown that many postmenopausal women consider the use of complementary or alternative medicine (CAM) for alleviating menopausal syndrome yet have concerns regarding its cost and efficacy [9]. Most women using CAM chose not to inform their physicians because they felt physicians were not good enough for judging the effects of CAM [10].

In postmenopausal women, vasomotor symptoms (VMS) including hot flush, sweating and palpitations are quite common [11]. Approved by the U.S. Food and Drug Administration (FDA), estrogen-based therapy remains the most effective for reducing VMS [12]. The indications of HRT include relieving VMS, treating menopause-associated atrophic vulvovaginitis and preventing osteoporosis. Furthermore, HRT elevates the mineral densities of bones and lowers the occurrence of fractures due to osteoporosis [13]. In consideration of the aforementioned harmful effects of HRT, it is recommended by the FDA to use the approved non-estrogen treatments carefully before using HRT solely for osteoporosis prevention [14].

Recently, nutraceuticals including phytoestrogens and herbal derivatives have drawn public attention because of their possible ability to alleviate menopausal symptoms [15]. For example, Actaea racemosa alleviates menopausal symptoms including hot flush, sweating, insomnia, irritability and musculoskeletal pain; Valerian officinal is effective for hot flush, anxiety and sleep disorders; Panax ginseng appears effective on treating depression, insomnia and improving sexual function; Ginkgo biloba can be used to improve attention disorders of postmenopausal women [15]. Unlike phytoestrogens, herbal derivatives such as Actea racemosa, Valerian officinal and Ginkgo biloba act via different estrogen-independent pathways to ameliorate menopausal symptoms.

Epidemiological research has explored a connection between the consumption of soybeans and reduced VMS [16]. The intake of soybeans has been estimated to be far greater in Asian countries including China, Japan, Taiwan and Korea than in Western countries. Relevantly, postmenopausal Asian women have significantly lower incidences (10–25%) of hot flush in comparison with those in Western countries (60–90%) [17].

Isoflavones can be extracted from soybeans. They can exert estrogen-like actions and thus alleviate postmenopausal syndrome. Two types of estrogen receptors include ERα, which is distributed predominantly in the uterus and breast, and ERβ, which is distributed predominantly in bone, the urogenital system and the cardiovascular system [10]. Generally, the binding affinity of isoflavones is higher to ERβ than to ERα. Using in vitro human cell culture for estimating the estrogenic effects of isoflavones, research has revealed the potencies as follows: estradiol (E2) 100, daidzein 0.013, genistein 0.084, and S-equol 0.061 [18]. Nevertheless, isoflavones may reach 10,000 times the E2 concentration and have significant stimulation due to their abundance [19]. An example showed the effect of isoflavones on female mice that were aromatase-knockout and estrogen-deficient. Isoflavone can act as estrogen and improve the ovarian morphology [20].

A variety of studies have reported the different effects of isoflavones and not reached an overall consensus yet. The possible explanations are that related trials vary in study designs and in the ingredients and doses of isoflavones and that trials are confined to small sample sizes and high rates of dropout [21]. Moreover, there is a high variability in age and onset of menopause for the recruited women. On the basis of prior studies discussing processing of soybeans, the nature of isoflavones and their mechanisms of action, the overview presents a summary of the current literature about processing of soybeans, isoflavone types (daidzein, genistein, and S-equol), supplements, extraction and analysis as well as information about the utilization of isoflavones in soybeans.

In this review, all of the reference articles were retrieved from the databases Medline and PubMed using the search term “isoflavone” for the topic. For further screening and selection, only full-text articles were considered to be included for a further analysis. A literature search was conducted on the Medline and PubMed databases up to 31 December 2020 to identify all potential articles in the data sources. In the screening process, duplicate articles and research before 1980 were excluded. After initial processing, two experts in the field independently reviewed potentially eligible studies for exclusion and inclusion by inspecting the sources including year, authors, research design and clinical outcomes. Articles with poor study design or unmatched outcomes were deemed not eligible for the study. During the period of study selection, disagreements between the two reviewers were resolved by mutual discussion until a consensus was reached. Using the search term and searching strategy (database identification, article screening, consideration of eligibility and final inclusion), all eligible articles were selected for the topic.

## 2. Processing of Isoflavones in Soybeans

### 2.1. Processing of Soybeans

**Figure 1:**
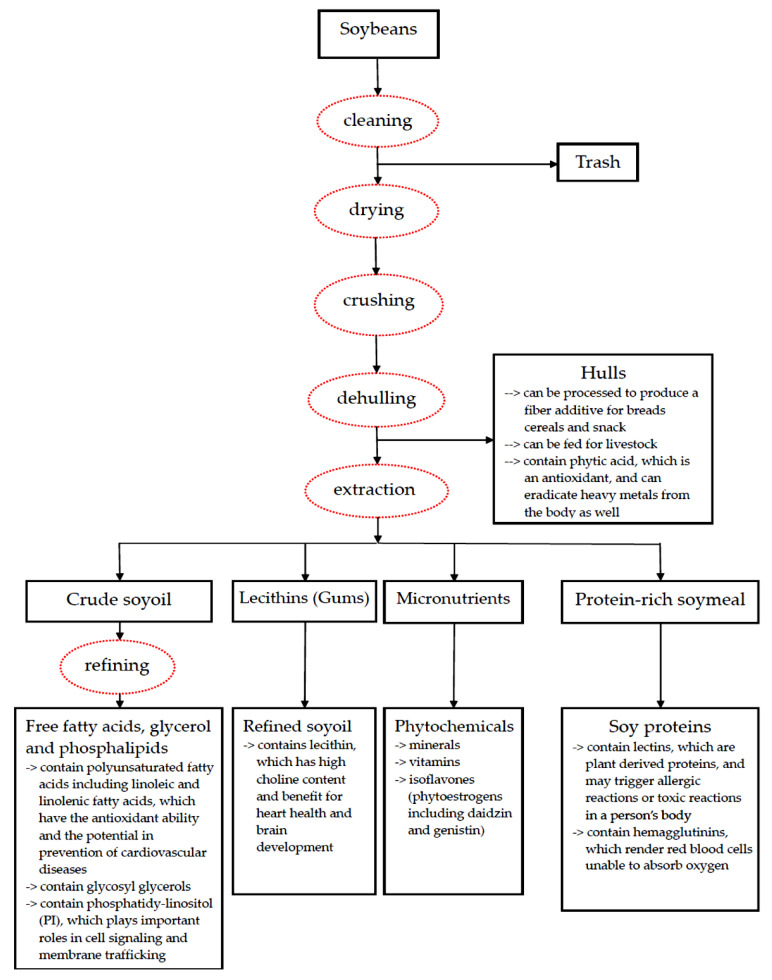
The processing, components and functions of soybeans.

Processing of soybeans refers to continuous steps to prepare soy products for use in aspects of food, industry and animal feed. Advanced processing can produce refined soy oil, glycerol, free fatty acids, lecithins (soy gums) and protein-rich soymeal containing soy protein concentrates and isolated soy proteins [1,2,3,22]. In addition, soybeans also contain a wide range of micronutrients and phytochemicals including minerals, vitamins, sterols and isoflavones. Isoflavones can be also extracted in the process for further use. The processing, preparation and extraction of soybeans are showed in Figure 1.

Before further processes are implemented, the soybeans need to be cleaned, dried (heated), crushed and then dehulled. The initial processing methods are as follows [1,2,3,22]:

(1) Cleaning: After a threshing operation, plant waste, small gravel, soil, insects, weed seeds and broken soybean seeds may contaminate soybeans. The cleaning procedure is performed several times in the post-harvest system.

(2) Drying: The operation involves drying the soybeans with a cycle of natural or artificial drying until the “safe-moisture” is reached.

(3) Crushing: The process consists of breaking the whole soybeans into pieces to facilitate dehulling.

(4) Dehulling: The process is to remove the outer covering from soybeans or other seeds.

As a major source of dietary fiber, the hulls (the tough exterior skin of soybeans) are processed to produce a fiber additive for breads, cereals and snacks. Livestock feed can also be manufactured from soybean hulls [1,2,3,22].

Even though there are continuous advancements and refinements in the technology of soybean processing, currently the main method used is referred to as the “extraction or solvent” process. Using organic solvents such as hexane, the method recovers 95–98% of the soy oil and 95% of the soy protein [22]. Typically, a bushel of soybean grains (60 pounds) can yield about 11 pounds (18.3%) of soy oil and 48 pounds (80%) of protein-rich meal. As for the rest, hulls make up the remaining 1.7% [22].

After extraction, crude soy oil can be obtained, but it has to be further processed to produce a refined oil. The additional processing can remove substances which compromise the quality of the soy oil. Another product of soy oil is soybean lecithin, which mainly consists of phospholipids and acts as an important natural emulsifying agent for food and pharmaceuticals [2,3,22]. Extracted from crude soy oil to avoid cloudiness and discoloration of the refined oil, lecithin can also be used as animal feed and in paints, lubricants and other industrial applications [1,2,3,22]. Glycerol and free fatty acids are formed as a result of soy oil breakdown. Free fatty acids are also used as emulsifying agents. Glycerol is collected from the oil under a deodorization process. As important nutritional substances, further products including sterols, sterol esters and the antioxidant tocopherol (vitamin E) are extracted from the oil as well under a deodorization process [1,2,3,22].

In addition, protein-rich soybean meal can be manufactured during the process, and it has been recognized as one source of proteins available for humans and livestock [2,3,22]. Soybean proteins are equivalent to animal proteins in quality, highly digestible and comparable to beef, fish, milk and egg proteins in terms of protein quality [22]. The detailed process of isoflavone extraction from soybeans is described in Section 2.3.

### 2.2. Isoflavones (Daidzin, Genistin, Daidzein, Genistein) and S-Equols

Isoflavones are composed of two benzene rings linked through a heterocyclic pyrane C-ring at the 3 position, which distinguishes them from flavones. Being malonyl-glucoside conjugates in chemical structures, the primary isoflavones in soybeans belong to the daidzein, genistein and glycitein families [3,4,7]. Each family comprises its respective aglycone, β-glucoside, malonyl-glucoside and acetyl-glucoside [3,4,7]. Malonyl-glucoside is the predominant form of many isoflavones in unprocessed soybeans. Research has revealed that malonyl-glucoside is heat sensitive and easily converted to its corresponding acetyl-glucoside and/or β-glucoside according to the thermal conditions of preparation and processing [7]. Similarly, a study has indicated that soy flour that had not been heat-treated mainly contained malonyl-β-glucoside conjugates; by contrast, heated soy flour consisted of large amounts of acetyl-β-glucoside conjugates, formed by means of heat-induced decarboxylation of the malonate group to acetate [4]. Analyzed using high-performance liquid chromatography (HPLC)-mass spectrometry, isolated soy proteins and textured vegetable proteins were composed of a mixture of all three types of isoflavone conjugates. Frying or baking of textured vegetable proteins at 190 °C and baking of soy flour did not change the total content of isoflavones, but there was a stable increase in β-glucoside conjugates at the expense of malonyl-β-glucoside conjugates [4]. Therefore, the chemical structures of isoflavones in foods should be considered when evaluating the availability of isoflavones for absorption from the diet [4]. Different processing conditions produce soybean products with a variety of isoflavone composition and content. Recently, research has shown that the chemical structures and abundance of isoflavones in soybean foods have an important impact on their biological effects and bioavailability [3,7].

A study has assessed isoflavone concentrations of various soybean cultivars with maturity grade 0 to 5 grown under different environments and analyzed the effects of other important seed characters [23]. Analyzed using HPLC-mass spectrometry, the components of isoflavones had abundant genetic variations in soybean seeds. Furthermore, individual and total concentrations of isoflavones in soybeans were significantly influenced by cultivar, site, year and maturity grading [23]. A trend existed in the content of isoflavones, in which isoflavone concentration was lower in earlier than in later maturing soybean cultivars. Nevertheless, a genetic factor still plays the most critical role for isoflavone concentration [23].

**Figure 2:**
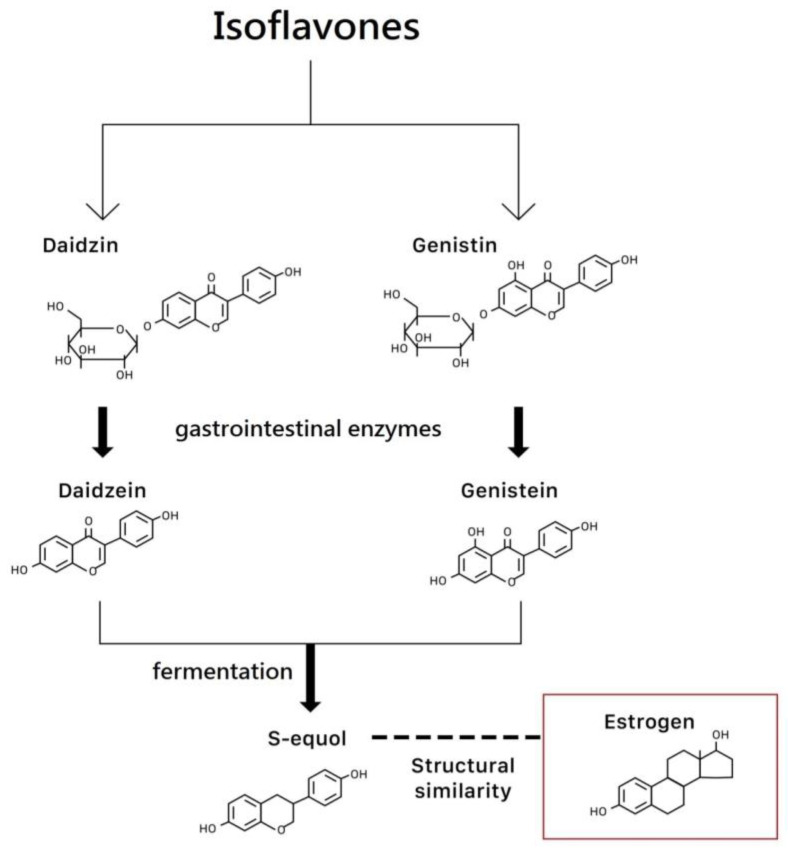
The chemical structures of isoflavones including daidzin and genistin and their derivatives daidzein, genistein and S-equol (structurally similar to estrogen).

Although the terms phytoestrogen and isoflavone are frequently used interchangeably in the literature, they are not exactly the same. More precisely, phytoestrogen is a plant compound that can exert an estrogen-like effect, and isoflavones, including daidzin and genistin, belong to bioflavonoids that are available in animals and plants [24]. Some foods such as soybeans, alfalfa and red clover contain abundant isoflavones. Through the metabolic actions of gastrointestinal enzymes in the human gut, both of the isoflavone precursors daidzin and genistin are transformed into daidzein and genistein, respectively [25] (Figure 2). The process of soybean fermentation concentrates isoflavone, while the process of removing the fat, color or taste predisposes the removal of isoflavone [26]. More than 50% of Asian women have intestinal bacteria that are capable of converting daidzein and genistein to S-equol, a bioactivator similar to estrogens in structure. However, this is not the case for the women on other continents [27] (Figure 2). Compared with isoflavones, S-equol has a similar preference for binding to ERβ but a higher transcriptional expression [26].

A study reported that even though non-equol producers consumed an adequate amount of daidzein, the effects of relieving menopausal symptoms were still limited [27]. A study enrolling a total of 364 women reported that equol producers gained benefits from daidzein for alleviating VMS [28]. Although ingestion of probiotics did not increase the production of S-equol [29], another trial which was conducted later indicated that the extract of red clover plus probiotics successfully improved VMS [19]. Equol-transforming bacteria such as *Lactococcus garvieae* were separated from the fecal cultures of equol-transforming women [29,30], and a randomized controlled trial (RCT) conducted in Japan revealed that a supplement with equol for non-equol producers effectively reduced postmenopausal emotional symptoms [31].

### 2.3. Extraction of Isoflavones from Soybeans

The concentrations of isoflavones are low in soybeans and related food products; therefore effective extraction prior to further analyses is quite important [3,7]. Before extraction, the preparation process includes removal of water from the samples and grinding or homogenization of samples [7].

Soybeans and related products contain high levels of proteins, lipids and carbohydrates. As a minor component of complex mixtures, isoflavone must be separated from the bulk of the matrix constituents prior to further analyses [3,7]. There is a practical way to extract isoflavones from soybeans in their original forms using available solvents and laboratory equipment [7]. According to chromatographic behaviors on reversed-phase columns in the presence of acids in the mobile phase to protonate the glycosidic isoflavones, the hydrophobicity of the isoflavone conjugates is aglycone > acetyl-glucoside > malonyl-glucoside > β-glucoside. The ester bonds of acetyl- and malonyl-glucose of isoflavones are liable to be broken under an elevated temperature and changed acidic or basic conditions [7].

The solvents used for extraction of isoflavones from soybeans and related foods can be chosen on the basis of the solubility of isoflavones and the soyfood matrix. Because of the diversity of soybean isoflavones in polarity, the extraction requires the combined use of organic solvents and water. The solvents, including acetone, acetonitrile, methanol and ethanol, have been used to extract isoflavones from soybeans and related foods [7]. Isoflavones are relatively steady compounds but can degrade under certain circumstances. Genistein and daidzein are light-sensitive [7]; malonyl-glycosides of isoflavones are heat-labile [4,7].

The extraction techniques used for bioactive components (isoflavones and other polyphenols) [6] and the summary (Table 1) are listed below.

#### 2.3.1. Maceration Extraction

Maceration is a conventional and very simple extraction method that uses different solvents for the extraction of components from soybeans or other plant material. Soybeans or other plant materials are left together with the solvent from a couple of hours up to several days, and after that, filtration procedures clean the extract from solid suspensions. It has the advantage of being a very suitable technique for thermally labile components.

The most used solvents for the extraction of the total amount of isoflavones, which are a kind of polyphenols, are methanol, acetone and water. Maceration extraction has proven to be an efficient technique for isolation of anthocyanidins, flavan-3-ols, proanthocyanidins, flavanones, flavones and flavonols subclasses when a mixture of 0.1% HCl in methanol and water (80:20, *v*/*v*) was used as a solvent. Ethanol is preferred to methanol due to its lower toxicity. Maceration extraction using 80% ethanol kept at room temperature for 7 days and the same solvent at 60–70 °C for 2 days has been applied to the extraction of other components.

#### 2.3.2. Soxhlet Extraction

Soxhlet extraction is the second conventional method used for bioactive component extraction, an improvement over maceration, as it uses solvent heating to the boiling point and the returning of the condensed vapors to the flask. In this way, Soxhlet extraction can be run over as many cycles as desired. Water and ethanol seem to be the most-used solvents in this extraction technique due to their availability, efficiency and non-toxicity. Polyphenolic-polysaccharide conjugates have been extracted using an Soxhlet apparatus.

#### 2.3.3. Ultrasound-Assisted Extraction (UAE)

UAE is an efficient extraction method at the border between classical and modern techniques and is used for a variety of bioactive compounds. Compared with maceration and Soxhlet procedures, UAE possesses several strategic advantages: Shorter extraction times, a reduced amount of necessary solvent and lower energy consumption are the most important benefits of UAE compared with conventional methods. The operating principle of UAE is the utilization of ultrasound (waves with a frequency between 20 and 100 MHz) to develop bubbles inside the solvent. These bubbles, once created by cavitation phenomenon, induce wall cells’ disruption of the targets and then speed up the penetration of the solvent into the target material. UAE using ethanol–water (1:1, *v*/*v*), with a solvent–sample ratio of 1:8, was applied to extract components in soybeans.

#### 2.3.4. Microwave-Assisted Extraction (MAE)

MAE is a well-established technique and is a promising alternative to conventional extraction techniques. It uses microwaves (non-ionizing electromagnetic waves with a frequency between 0.3 and 300 GHz). The absorption of microwaves by polar molecules (such as water) and, implicitly, the heat generation, facilitates diffusion of the solvent into vegetal samples and release of the solutes from the target material into the solvent. The advantages of MAE over the previously presented techniques are considerable savings in time, solvent amount and energy consumption and therefore an increase in extraction efficiency. MAE using water, methanol–water (80:20 *v*/*v*), ethanol–water (80:20 *v*/*v*) and time/temperature programs were applied for extraction of polyphenols including flavones.

#### 2.3.5. Supercritical Fluid Extraction (SFE)

Supercritical fluid extraction is a valuable and environmentally friendly extraction technique used for extracting a large variety of bioactive compounds, presenting the advantages of being fast, selective and solvent saving. A supercritical state occurs when the temperature and pressure of the fluid are raised above its critical point. Carbon dioxide is the most used solvent in SFE, being very efficient for extraction of fat, lipids and other non-polar compounds. For extraction of polar substances, a polar modifier called co-solvent (for example, methanol, ethanol, acetonitrile, acetone, water, ethyl ether or dichloromethane) is necessary to be added to the supercritical fluid in order to increase the solubility.

#### 2.3.6. Accelerated Solvent Extraction (ASE)

Accelerated solvent extraction is a modern extraction technique used for the recovery of bioactive compounds involving solvents under high temperature and pressure but without reaching the critical point. In this extraction method, different solvents are required; the most popular are methanol, ethanol or mixtures of other solvents, as well. The 50% acidified methanol (50MA) followed by 50% acidified acetone (50AA) was the most efficient solvent for the extraction of phenolic compounds, particularly flavonols, while water (W) was not beneficial to the extraction of all categories of phenolics. ASE was more suitable for the extraction of phenolics compounds than UAE.

### 2.4. Analyses of Isoflavones Using HPLC

HPLC can be used for analyses of isoflavones in soybean and related products. HPLC is a fast, reproducible method requiring small sample sizes and is eligible for both qualitative and quantitative analyses as well as for the purpose of sample separation. With an authentic isoflavone standard available, the appropriate method to analyze isoflavone extracts from soybeans is usually HPLC coupled with a reversed-phase C18 column (silica-based packings with covalently bound 18-alkyl chains) and UV spectrophotometer [7]. According to the solubility and polarity of isoflavones in the column between the stationary and solvent (mobile) phases used, mixed isoflavones in soyfood extracts can be isolated. Each isoflavone exhibits respective intense absorption in the UV region of the spectrum ranging from 240 to 280 nm [7]. In addition, using a C18 HPLC column and gradient solvent phase, a reversed-phase separation can be achieved to isolate isoflavones in the soyfood extract. Reversed-phase HPLC acts based on lipophilicity and hydrophilicity [7].

## 3. Utilization of Isoflavones for Menopause-Related Syndromes and Others

### 3.1. Vasomotor Syndromes (VMS)

Vasomotor syndromes (VMS), such as hot flush and sweating after menopause, are usually the most troublesome discomforts for affected women, and most of the women will look for medical help. Nevertheless, because the severity and frequency of hot flush and sweating are individualized, and such symptoms often subside over time even without any treatment [25], it is difficult to quantify VMS. In 2001, a study conducted by St. Germain et al. revealed that hot flush declined in all of the patients who respectively took isoflavone-containing soybeans, isoflavone-free soybeans or whey proteins over 24 weeks, indicating that a placebo effect was inevitable [32]. Likewise, another study reported that the occurrence of hot flush did not differ between the women using isoflavones and those using placebos over 12 weeks [33].

However, most recent studies favor using isoflavones as a treatment of VMS. A small-scale prospective study reported a 57% reduction in the severity and frequency of hot flush for menopausal women who took 60 mg isoflavones daily over 12 weeks [34]. Using the Kupperman Menopause Index, Cancellieri et al. also reported that 72 mg of isoflavones retrieved from soybeans and red clover significantly decreased hot flashes over 6 months of treatment [35].

In contrast to the Western diet, soy nuts were given for 8 weeks as a substitute for non-soy protein, and this study conducted by Welty et al. noted a >40% reduction in the symptom of hot flush in menopausal women, regardless of equol-producing status [16]. The results of this study provided an option for Western women to obtain the benefits of isoflavone by eating an Eastern diet. Furthermore, a systematic review reported that synthetic isoflavone may have been more effective in reducing the symptom of hot flush compared with soy isoflavone [36].

Another focus is the optimal dosage of isoflavone for symptomatic women after menopause. A report conducted in 1999 by Washburn et al. revealed that compared with administrating the total amount of soy supplement in one dose, dividing the dose into twice daily better reduced the severity of hot flush, suggestive of a better effect of phytoestrogens with consistent circulating levels [37]. Another study reported a similar finding in that both 40 mg and 60 mg of the daidzein-containing isoflavone aglycone given once daily could reduce the frequency of hot flush by the same degree [38].

Researchers have studied the synergistic action of inulin plus isoflavone. Focusing on peri-/post-menopausal women treated or untreated with a mixture of soy isoflavone (40 mg), inulin (3 g), vitamin D3 (300 IU) and calcium (500 mg), a prospective multicenter study reported the effect of inulin and soy isoflavone on hot flush and life quality [39]. The mean number of hot flushes was found to decline more for women in the treated group compared with those in the untreated group. The results of the study revealed the benefits of using a combination of inulin and soy isoflavone as a dietary supplement for women with menopausal symptoms [40].

Nonetheless, isoflavone is still less effective when compared with traditional HRT. Isoflavone 45 mg twice daily, low-dose HRT (E2 1 mg and norethisterone acetate 0.5 mg) and a placebo have been compared in a RCT. The results showed that isoflavone and low-dose HRT were more effective than the placebo, yet low-dose HRT was better than isoflavone [40]. Making indirect comparisons, another report analyzing more than 50 RCTs disclosed the difference of effects between soy extracts and HRT for relieving hot flush [41]. However, over a research period of 24 months, another study reported that an isoflavone mixture containing daidzein 22.01 mg, glycitein 13.54 mg and genistein 4.96 mg did not affect quality of life as measured with Menopause-Specific Quality of Life (MENQOL) [42]. The result of the study mentioned above revealed that isoflavone cannot entirely replace the role of traditional HRT in relieving symptoms for menopausal women.

Research has revealed that whether or not isoflavone can effectively reduce VMS depends on the ability to produce S-equol in women. A report analyzing several RCTs investigated the effects of S-equol and isoflavone to alleviate VMS in women who were equol-producers or non-producers [43], and the results of the meta-analysis showed a significant benefit of equol for lower hot flush scores. Therefore, the meta-analysis concluded that the supplementation of equol remarkably decreased the occurrence and severity of VMS in postmenopausal non-producers [43].

Some studies have used skin conductance measurement to avoid subjective evaluation of the incidence and severity of hot flush. Skin conductance measurement can quantify a tiny change in individual core temperature as well as later reaction [19]. A study conducted by Newton showed consistency between the use of a skin conductance monitor and a diary [28], while Lambert et al. reported inconsistency between hot flush measurement using all-day ambulatory skin conductance and a subjective report of hot flush with the Green Climacteric Scale [19]. The latter study revealed that red clover extract significantly decreased the incidence of hot flush that was measured by skin conductance rather than by a scale, thus emphasizing the importance of measurement with objective instruments [19].

### 3.2. Hormone-Related Osteoporosis

After menopause, another noteworthy change is a gradual loss of bone mineral density (BMD), resulting in osteoporosis. Because expression of ERβ is higher in bone tissues [24], theoretically, isoflavone can alleviate loss of bones. A prior study investigated the effect of ipriflavone, one of the synthetic isoflavones; however, the results were been inconclusive [25].

There is a wider distribution and stronger expression of ERβ in trabecular bone [44]; hence, the spine is deemed the site most sensitive to isoflavone due to a higher content of trabecular bone than cortical bone. In contrast, the hip consists of more cortical bones, and thus undergoes slower remodeling compared with the spinal bones [45]. In a report of a meta-analysis, spinal bone loss was significantly alleviated after a daily 90 mg isoflavone supplement for 6 months [45]. A study conducted by Amato et al. revealed that although daily 120 mg isoflavone failed to prevent loss of local bones, systemic BMD loss was attenuated [42]. A meta-analysis reported in 2017 mentioned that isoflavone alleviated BMD loss, but more prominently at the lumbar spine than the femoral neck, and the form of isoflavone in aglycone was even more efficacious [44].

Because bone remodeling needs from 4 to 8 months, research with a longer duration could assist in elucidating a more conclusive result [44]. Meanwhile, for postmenopausal women without hormone replacement therapy, even a slight improvement in BMD can be beneficial [25].

### 3.3. Female Urogenital System

Research has identified the variable distribution of both ERα and ERβ in female urogenital organs such as the vagina, urethra and bladder [46]. In addition, the aforementioned organs contain GPERs, which possess a greater affinity for daidzein and genistein. In the past, topical estrogen was applied to vaginal and urethral surfaces for treating vaginal dryness and urinary incontinence; however, the detailed mechanisms of improving incontinence remain unknown. Currently, isoflavone is being tried for the treatment of urogenital symptoms after menopause. However, a longitudinal study over a 10-year duration revealed that neither higher nor lower intake of dietary isoflavone could prevent urge or stress incontinence [46], though equol-producer status was not considered. On the other hand, Burton and Wells have not found any substantial evidence to confirm that phytoestrogen affects the female genital system, but this can be attributed to a lack of research rather than a paucity of correlation and urges further investigation into this issue [47]. A study reported no difference in the cytology of the vagina for women who used dietary soy or black cohosh for one year [48], although the percentage of isoflavone in the supplement was not clearly calculated.

A prospective, randomized study identified the effect of nutraceuticals on the sexual function of postmenopausal women. This trial evaluated a combined use of isoflavone (40 mg), inulin (3 g), vitamin D (300 IU) and calcium (500 mg) in postmenopausal status [49]. The researchers found that patients in the treatment group had better scores in the physical and vasomotor domains of life quality evaluation and better scores in all domains of sexual function evaluation after 12 months, suggestive of a favorable effect of the combination of isoflavone, inulin, vitamin D and calcium on life quality, menopausal symptoms and sexual function [49].

Recently, a randomized clinical trial was conducted on 60 postmenopausal women, who were randomly assigned to receive oral isoflavone (150 mg dry extract of glycine max) alone or isoflavone plus probiotic or hormone therapy (1 mg estradiol and 0.5 mg norethisterone acetate) to evaluate genitourinary symptoms and vaginal atrophy [50]. The result revealed that after 16 weeks of treatment, the urogenital symptoms including vaginal dryness and complaints of sexual problem as well as the maturation value, vaginal pH and vaginal flora improved significantly in the hormone therapy group. There was a significant increase in daidzein, glycitein, equol intermediate, and O-dimethylangolensin contents after 16 weeks in the isoflavone plus probiotic group. The vaginal health score increased in the isoflavone and hormone therapy groups. Therefore, it was concluded that probiotics improved the metabolism of isoflavones after 16 weeks of treatment. However, the increase in the contents of isoflavones and their metabolites failed to yield an estrogenic effect on the urogenital tract and relieve vulvovaginal symptoms [50].

### 3.4. Metabolic Syndrome

After menopause, relative changes of metabolism can lead to obesity, which is a major risk factor for cardiovascular events [51]. Current evidence obtained from one study supported that the use of isoflavone can ameliorate glycemic control and facilitate weight loss [52]. However, there are limited studies researching the relationship between soy isoflavone supplementation and body weight loss in obese menopausal women. Although another study has shown that isoflavone and exercise can result in a reduction of fat mass, its conclusion is somewhat questionable due to a high dropout rate and poor patient compliance to treatment [51].

An in vitro study showed that daidzein, genistein and equol all had good binding affinity to activate the peroxisome-proliferator-activated-receptor (PPAR)γ, which played a key role in many metabolic syndromes and diabetes [52]. In comparison with rosiglitazone, which was a well-known drug for diabetes, the maximal isoflavone-induced PPARγ activity was between 23% and 32% [52]. Because soybeans rarely induced gains in body weight as rosiglitazone did [52], isoflavone might be considered for the treatment of metabolic syndromes.

### 3.5. Cardiovascular Disease

The etiology and pathophysiology of cardiovascular diseases are complex and multifactorial, and cardiovascular events increase in postmenopausal women because of aging and estrogen deficiency [53]. A paucity of estrogen brings about elevation in cholesterol and low density lipoprotein and thus endothelial dysfunction [54]. Acting as an estrogen substitute, isoflavone may be capable of reducing the risk of cardiovascular events.

Soy products are generally deemed healthy foods due to nearly no cholesterol content, and therefore the FDA has stated that soybean proteins may lower the risks of cardiovascular diseases [25]. Nevertheless, there is habitually a low intake of soybeans in Western countries. Interestingly, another study reported no connection between a habitual phytoestrogen intake (including isoflavone and lignan) in the Western-style diet and the risk of cardiovascular diseases, possibly due to an overall low intake of isoflavone [53].

The detailed mechanisms of isoflavone to prevent cardiovascular diseases remain unclear. Previously, a study showed the benefits of isoflavone in ameliorating systemic arterial compliance of perimenopausal and postmenopausal women, even if it did not influence plasma lipids [54]. Similarly, a study conducted in 2007 revealed no difference in lipoprotein levels after using 60 mg isoflavone per day for 12 weeks [34]. Hence, isoflavone may exert its protective effect on cardiovascular diseases in other ways [55].

Daidzein and genistein have been thought to cause arterial relaxation by means of releasing nitric oxide [55]. Nonetheless, a study conducted by Wong et al. showed no significant difference in blood pressure after using daily 80 mg soy isoflavone for 6 weeks [56]. In contrast, animal research has indicated a deleterious effect of soybeans or HRT in vessels with a preceding change of atherosclerosis. Neither soybeans nor HRT could reduce the incidence of myocardial ischemia or reperfusion injuries in ovariectomized or diet-induced atherosclerotic monkeys. Furthermore, a combination of soybeans and HRT practically increased myocardial changes after local ischemia [57]. However, the inferences from animal research must be made with caution. Before suggesting that women avoid consumption of soybean products once they have cardiovascular events, more studies are required in the future to draw a reliable conclusion [55].

Recently, an RCT indicated that isoflavone in soybeans could reduce systolic blood pressure in the early stages of menopause. Sathyapalan et al. noted that supplementation with soy isoflavone for 6 months could reduce systolic blood pressure in women who were menopausal for less than 2 years, although both diastolic blood pressure and lipid profiles (triglycerides, HDL, LDL and total cholesterol) remained unchanged [58]. Using the Framingham equation, a reduction of risk in cardiovascular events can be calculated using personal blood pressure [58]. Based on the findings mentioned above, it can be inferred that the best and safest treatment window for cardiovascular diseases will be early menopausal stages and before critical atherosclerotic changes occur.

Isoflavone probably only plays a minor role in the prevention of cardiovascular diseases, and the major benefits disclosed are from soybeans themselves. Compared with animal proteins, soybeans contain minimal saturated fats and cholesterol [25] and subsequently make it healthier to meet daily nutritional requirements. Hence, the American Heart Association and FDA recommend soybean foods for their benefits to overall and cardiovascular health [25,51] whether the rationale is based on soybeans containing copious amounts of fibers, vitamins and polyunsaturated fats [51] or simply on the fact that they replace a dietary intake of animal proteins [26].

Recently, a study investigated the health benefits of soy products for the reduction of coronary heart disease (CHD). A total of 74,241 women from the Nurses’ Health Study (1984–2012), 94,233 women from the Nurses’ Health Study II (1991–2013) and 42,226 men from the Health Professionals Follow-Up Study (1986–2012), who were free of cardiovascular disease and cancer at baseline, were included [59]. In multivariable-adjusted analyses, isoflavone intake was inversely associated with CHD (pooled hazard ratio (HR) comparing the extreme quintiles: 0.87 (95% CI 0.81–0.94); *p* < 0.01). Consumption of tofu, but not soy milk, was inversely associated with the risk of CHD, with pooled HRs 0.82 (95% CI 0.70–0.95; *p* < 0.01) and 0.87 (95% CI 0.69–1.10; *p* = 0.41), respectively. The study concluded that higher intake of isoflavones and tofu was associated with a moderately lower risk of developing CHD [59].

### 3.6. Cognitive Function and Neuromuscular Systems

Although the natural aging process is inevitably a confounding factor, research has still explored the effect of isoflavone on the cognitive function of postmenopausal women. Research enrolled menopausal populations with various intakes of isoflavones over 6 years and found that perimenopausal and postmenopausal women in Asia with a higher intake of isoflavone had better function in processing speed but worse function in verbal memory [60]. However, the overall effect of isoflavone on cognition was small, thus inducing doubt about the importance of the above findings. A meta-analysis reviewing 12 RCTs reported that isoflavone and soybeans might improve the cognitive function of menopausal populations; however, several obtained RCTS had problematic methods and designs to some extent [61]. In 2011, the North American Menopause Society (NAMS) reported the benefits of soybeans in the function of cognition for postmenopausal women aged younger than 65 years old, yet not for those older [26].

A cross-sectional study evaluated the associations between intake of soy products and isoflavones with depressive symptoms in 1745 pregnant Japanese women [62]. The result revealed that higher intake of total soy products, tofu products, fermented soybeans, boiled soybeans, miso soup and isoflavones was independently related to a lower prevalence of depressive symptoms during pregnancy: The adjusted prevalence ratios (95% CI) between extreme quartiles were 0.63 (0.47–0.85), 0.74 (0.56–0.98), 0.57 (0.42–0.76), 0.73 (0.55–0.98), 0.65 (0.49–0.87) and 0.63 (0.46–0.86), respectively. A significant positive exposure–response relationship was found between miso intake and depressive symptoms during pregnancy. No material relationship was observed between soymilk intake and depressive symptoms during pregnancy. The study showed independent inverse relationships between intake of soy products and isoflavones and depressive symptoms during pregnancy [62].

A study explored whether the dietary isoflavone aglycone (AglyMax) at 0.6% could prevent denervation-mediated muscle atrophy based on the modulation of atrogin-1- or apoptosis-dependent signaling [63]. Mice were fed either a normal diet or 0.6% AglyMax diet, and then the right sciatic nerve was cut for examination. The 0.6% AglyMax diet significantly attenuated denervation-induced decreases in fiber atrophy but not the muscle wet weight. In addition, dietary isoflavone suppressed denervation-induced apoptosis. The authors concluded that the isoflavone aglycone significantly modulated muscle atrophy after denervation in mice, probably due to the decrease in apoptosis-dependent signaling [63].

### 3.7. Adverse Effects

Generally, isoflavone was well-tolerated by human bodies in all of the studies available for this review. Adverse effects were usually mild and mainly gastrointestinal such as bloating, nausea, constipation and diarrhea [8].

Previous results of animal studies have raised concerns about S-equol because infertility and reproductive anomalies have been noted among animals fed with soybean-containing diets or red clover [29]. In mice undergoing oophorectomies, feeding with a high dose of equol for 3 months induced obvious mammotropic effects [64]. Nevertheless, the use of S-equol seems safe for the uteri of humans among all of the studies available.

Since estrogen is likely to contribute to the differentiation of male external genitalia, dietary intake of isoflavone, which has a similar structure to human estrogen, may be associated with the occurrence of hypospadias. A nationwide birth cohort study recruited women in Japan between 2011 and 2014 [65]. Among 41,578 mothers who delivered singleton, live male births, the median genistein intake was 15.3 mg/day, and 51 cases of hypospadias were identified. Compared with mothers in the reference group (genistein intake 11th–89th percentiles), those in the low-intake group (≤10th percentile) had an elevated risk of hypospadias in their sons (multivariable-adjusted OR = 2.8, 95% CI 4–5.8). Adverse or beneficial effects of genistein on hypospadias were not observed in the high intake group (≥90th percentile) (OR = 0.9, 95% CI 0.4–2.4). It is concluded that low maternal intake of isoflavone in early pregnancy was associated with an elevated risk of hypospadias [65].

### 3.8. The Risks of Cancers

Isoflavone can bind to estrogen receptors, thus raising concerns about its potential to induce estrogen-dependent malignancies, especially in the population of survivors of breast malignancy [51]. Nonetheless, research has indicated proliferation of breast cancer depends on the activity of ERα, while ERβ activation appears to suppress proliferation of ERα-induced cancer cells [66]. Hence, isoflavone is postulated to protect breasts from cancers by binding to ERβ. In addition, phytoestrogen has been noted to prolong the menstrual cycle, which is a possible reason for its protective effects against hormone-sensitive malignancies [25]. However, a detailed mechanism has not been fully clarified.

Focusing on healthy women, research has shown a decreased incidence of estrogen-related cancer in women using isoflavone. In Japan, a case–control study revealed that consumption of soybeans was inversely associated with the risk of breast cancer in women before menopause but not in those post menopause [67]. In a 12-week study, although soy isoflavone elicited some benign ultrasonographic changes of the breast, all of these changes were subclinical and did not need further intervention or follow-up after the study [68]. Moreover, soy isoflavone did not appear to stimulate proliferation of endometrial cells if given over a short term [26]. On the contrary, Shike et al. conducted an RCT to explore the effects of soybean intake on the gene expression of breast cancer. In their study, it was noted that supplementation with soybeans was related to enhanced gene expression, and there was a connection between high plasma levels of genistein and over-expression of FGFR2 genes to activate the proliferative pathway. Hence, they concluded that soybeans could unfavorably affect gene expression of breast cancer cells in a subset of women [69].

A meta-analysis aimed to systematically evaluate and summarize available evidence on the effect of dietary isoflavone intake on the risk of developing breast cancer [70]. PubMed, Embase, and the Cochrane Library were searched for prospective cohort studies that evaluated the effect of dietary isoflavone intake on the development of breast cancer. Among 16 studies, 11,169 breast cancer cases and 648,913 participants were identified and included in the data analysis. The pooled relative risk (RR) of breast cancer was 0.99 for high versus low intake of isoflavones (95% CI 0.91–1.09; *p* = 0.876) and 0.99 for moderate versus low intake of isoflavones (95% CI 0.92–1.05; *p* = 0.653). While a moderate consumption of soy-based foods did not significantly affect breast cancer risk, a high intake of soy-based foods was associated with a lower risk of developing breast cancer. The present meta-analysis indicated that women with a high dietary intake of soy foods might experience a statistically significant reduction in breast cancer risk [70].

Limited research has explored the role of soy isoflavone in postmenopausal patients who had breast cancer and underwent surgeries. The study conducted by Kang et al. revealed that a higher intake of soy isoflavone was related to a lower risk of breast cancer recurrence in the women who had positive ERs and progesterone receptors (PRs) and used anastrozole after surgeries [71].

A study attempted to evaluate the association of soy food or isoflavone intake with endometrial cancer risk in Japanese women [72]. This prospective cohort study enrolled 49,121 women aged 45–74 years, among whom 112 newly diagnosed endometrial cancer cases were identified. Cox proportional hazards regression models revealed that energy-adjusted intakes of soy food and isoflavone were not associated with a risk of endometrial cancer. Therefore, there was no evidence of a harmful association between soy food or isoflavone intake and endometrial cancer risk [72].

Another study aimed to determine whether long-term isoflavone soy protein (ISP) supplementation affected endometrial thickness and rates of endometrial hyperplasia and cancer in postmenopausal women [73]. In this randomized, double-blind, placebo-controlled trial, women aged 45 to 92 years were randomized to a total daily dose of 154 mg in the ISP group or a milk protein-matched placebo. During a 3-year period, a total of 224 participants were evaluated. The treatment group did not significantly differ from the mean baseline in endometrial thickness. Although the rate of hyperplasia/malignancy was higher in the placebo group (14.3% vs. 0%), the difference was not statistically significant. The study concluded that 3-year ISP supplementation had no effect on endometrial thickness or on the rates of endometrial hyperplasia and cancer in postmenopausal women [73].

Furthermore, a prospective study analyzed 46,027 non-hysterectomized postmenopausal women who were recruited into the Multiethnic Cohort (MEC) Study [74]. Among these women, 489 women diagnosed with endometrial cancer were identified during a median follow-up period of 13.6 years. Cox proportional hazards models revealed a reduced risk of endometrial cancer was associated with total isoflavone intake (highest vs. lowest quintile, relative risk (RR) = 0.66, 95% CI = 0.47–0.91), daidzein intake (highest vs. lowest quintile, RR = 0.64, 95% CI = 0.46–0.90) and genistein intake (highest vs. lowest quintile, RR = 0.66, 95% CI = 0.47–0.91). This study suggests that greater consumption of isoflavone-containing foods is associated with a reduced risk of endometrial cancer in this population of non-hysterectomized postmenopausal women [74].

A population-based prospective study evaluated the associations of soy and isoflavone intakes with bladder cancer incidence in Japan and enrolled 14,233 men and 16,584 women aged 35 years or older [75]. During a mean follow-up of 13.6 years, 120 men and 41 women had developed bladder cancer. After adjustments for multiple confounders, compared with the lowest quartile of soy food intake, the estimated hazard ratios for the second, third and highest quartiles of soy food intake in men were 0.74, 0.52 and 0.55, respectively. The corresponding values in women were 0.60, 0.75 and 0.64, respectively. Similar inverse associations were observed between isoflavone intake and bladder cancer risk. A significant decreased risk of bladder cancer was observed among individuals who had higher intakes of total soy and isoflavones. The study explored the potential benefit of consuming soy foods and isoflavones against bladder cancer [75].

In addition, isoflavone has also played a role in protecting against colorectal cancer. Isoflavone treats diabetes, hyperlipidemia and obesity, all of which are potential risk factors for colorectal cancer. Hence, it would be a reasonable hypothesis to state isoflavone prevents colon cancer. In Korea, a study reported that a high intake of isoflavone or soy foods was related to an overall reduced risk of colon cancer, though an over-ingestion of fermented soyfoods appeared to elevate the risks of male colon cancer, perhaps owing to their salty contents [76]. Future investigations are still needed for confirmation of this hypothesis. Other than isoflavone, soy fibers may also have a role in the prevention of colorectal cancer and warrant a comparison between soybeans and isoflavone rich extracts in the future.

Table 2 is a brief summary showing current evidence of studies regarding the preventive and therapeutic effects of isoflavones and soy foods on menopausal syndromes and other diseases.

## 4. Discussion

Regarding the preventive effects on diseases or cancers, a higher intake of isoflavones and tofu is associated with a moderately lower risk of developing coronary heart disease. Moreover, a high dietary intake of isoflavones or soyfoods may reduce the risks of breast and colorectal cancer as well as the incidence of breast cancer recurrence. Consumption of isoflavones and soy products is associated with a reduced risk of endometrial and bladder cancer. Regarding the therapeutic effects on menopausal syndrome or other diseases, isoflavones and soy extracts are superior to placebo in relieving vasomotor syndrome, but appear less effective than HRT. Use of isoflavones increases spinal BMD and ameliorates hypertension and in vitro glycemic control. Soy products and isoflavones may alleviate depressive symptoms during pregnancy, yet they fail to relieve vulvovaginal symptoms and stress or urge incontinence.

Because the concentrations of isoflavones are low in soybeans and related products, effective extraction prior to further analyses is quite important. Before extraction, the originals should be well prepared during the process, which includes cleaning, removing water from the samples and grinding. Different processing conditions produce soybean products with a variety of isoflavone composition and content. The chemical structures and abundance of isoflavones in soybean foods may have an important impact on their biological effects and bioavailability.

In general, the findings of previous studies are highly heterogenic, and it is quite difficult to draw conclusions regarding the effects of isoflavones [77,78,79]. Since 2011, the NAMS has recommended in a considerable way the prescription of isoflavone for menopausal syndrome. The recommendation is to initiate the use of isoflavone at a higher dose (more than 50 mg/day) for twelve weeks and monitor potential side effects. However, the treatment should be stopped in case of no response after a 12-week treatment [26]. In some women with menopausal symptoms, isoflavone treatment was considered relatively safe and beneficial. Another report also deemed isoflavone as an acceptable alternative for the menopausal population who suffered VMS and did not use HRT [80].

The physiological pathways of soybeans and isoflavones as well as their derivatives are not fully understood, as well as the interactions between these compounds and the human body. A previous in vitro study has shown that isoflavone can function as an agonist of estrogens after menopause due to a low-estrogen environment. In contrast, isoflavone can play the role of an antagonist of estrogens before menopause because of a higher circulatory level of endogenous estrogen [10]. Nevertheless, many questions still remain unanswered. Currently, it is not known which component of isoflavone produces the estrogen-like effect. Previous research has reflected this condition by examining a variety of isoflavone extracts and different parts of soybeans [81]. It is probable that substances in soybeans other than isoflavones can exert a favorable effect as well. It is thought that a wide range of health benefits from soy phytochemicals (lipids, fatty acids, soy sterols, vitamins, minerals and soyasaponins) contributes to the overall nutritional well-being of humans. The result was similar in another study where isoflavone has been removed from soybean products and compared with soybean extracts containing isoflavone [82]. Furthermore, a systematic review showed that synthetic isoflavone was superior to soybean isoflavone in alleviating hot flush [83]. In summary, the current evidence suggests that soybeans, isoflavones and their derivatives may function as an alternative via both estrogen-dependent and independent pathways [26].

There are some controversies regarding the trials to standardize the level of isoflavone for relieving menopausal symptoms in Asian women. A study reported hot flush occurred less in women of Eastern countries irrespective of soybean ingestion yet admitted that their equol-producing status was not fully considered [17]. Burton and Wells found uterotrophic effects in rats with neonatal exposure to phytoestrogen [47]. On the other hand, another human study recognized early-life exposure to dietary isoflavones as a precondition of the effects, suggestive of a possible early transformation of genes [26]. Because of the unpredictability in the action and response of isoflavone, an optimal dose of isoflavone remains to be determined. A study disclosed the response to S-equol exposure was approximately linear with dose, and a twice-daily dose might be better [27]. A previously study indicated that the total amount of isoflavone, estimated in aglycone-equivalent weight, was possibly 40–50 mg/day for symptom relief in women [83]. However, there was no study suggesting the upper limit for isoflavone use. Utian et al. reported twice per day instead of quotidian use of S-equol had a better effect [84]. Likewise, Crawford and his colleagues reported that isoflavone given twice per day could provide greater relief of hot flush than daily use, but the benefit of doubling the dose may have been for equol producers only [85]. Compared with these studies, a report did not show a linear but instead a biphasic effect of glabridin, a different isoflavone on the Ishikawa cell [86]. Therefore, daidzin, genistin, S-equol and other isoflavones appear idiosyncratic in their respective actions on humans and should be investigated individually. The limitations in previous studies warrant further investigations of isoflavone use for women’s health.

## 5. Conclusions

The previous studies that elucidated the structures and functions of bioactive molecules in soybeans have provided an insight into correlation between the reported health benefits associated with soy intake and soy phytochemicals. The processes of preparation (cleaning, drying, crushing and dehulling) and extraction of soybeans are implemented to produce refined soy oil, soy lecithin, free fatty acids, glycerol and soybean meal. Because the concentrations of isoflavones are low in soybeans and related products, effective extraction prior to further analyses is very important. Different processing conditions produce soybean products with a variety of isoflavone composition and content. Isoflavones including daidzin and genistin and their derivatives daidzein, genistein and S-equol (structurally similar to estrogen) can act to affect human endocrine functions. The chemical structures and abundance of isoflavones in soybean foods may have an important impact on their biological effects and bioavailability.

To summarize the therapeutic effects on menopausal syndrome or other diseases, isoflavones appear to reduce hot flush and other VMS even after accounting for a placebo effect. They may alleviate lumbar spine loss, improve BMD and have a positive effect on the control of blood pressure and sugar during early menopause. They may also ameliorate depressive symptoms during pregnancy. Currently, research has not drawn consistent conclusions regarding the benefit of isoflavone on the cognitive and urogenital systems. Regarding the preventive effects on diseases or cancers, a higher intake of isoflavones is associated with a moderately lower risk of developing coronary heart disease. It may also reduce the risks of breast and colorectal cancers as well as the incidence of breast cancer recurrence. Consumption of isoflavones or soy foods is associated with reduced risks of endometrial and bladder cancer.

Even though isoflavones are not as effective as traditional HRT in alleviating menopausal syndrome, a previous survey reported that 70% of women were satisfied with a non-hormonal intervention that decreased VMS by more than half [79]. The relative safeness of isoflavone as well as its benefits to overall health make it a considerable option for the treatment of postmenopausal women unable or unwilling to use HRT.

To minimize the heterogeneity of future studies, standardization of some influential factors involved in isoflavone research should be considered. One of the influential factors is “time to menopause”, which has an important impact on the individual response to isoflavones. The aglycone ratio in isoflavones needs to maintain consistency among future studies for a superior comparison of the therapeutical effects and to ensure reliability. Moreover, study duration and outcome measures also need standardization together with key endocrinal profiles of affected women. Furthermore, a larger sample sizes are required to draw reliable conclusions and improve the efficacy and replicability of study results.

## Figures and Tables

**Table 1:** A summary of extraction techniques used for bioactive components (isoflavones and other polyphenols).

Methods	Descriptions	Solvents	Advantages
Maceration extraction	Soybeans are left together with the solvent from hours up to days for maceration, and after that, filtration procedures clean off the extract from solid suspensions.	methanol acetone ethanol water	1. very simple; 2.very suitable technique for thermally labile components
Soxhlet extraction	It uses solvent heating to the boiling point and the returning of the condensed vapors to the flask. This way, it can be run for as many cycles as desired.	ethanol water	1. simple; 2. low cost
Ultrasound-assisted extraction (UAE)	It utilizes ultrasound to develop bubbles inside the solvent. These bubbles created by cavitation phenomenon induce wall cells’ disruption of the targets and speed up the penetration of the solvent into the target material.	ethanol water	1. shorter extraction time; 2. a reduced amount of necessary solvent; 3. lower energy consumption
Microwave-assisted extraction (MAE)	It uses microwaves, which can be absorbed by polar molecules (such as water), and facilitates diffusion of the solvent into vegetal samples and release of the solutes from the target material into the solvent.	Methanol ethanol water	1. considerable savings in time, solvent amount, energy consumption; 2. an increase in extraction efficiency
Supercritical fluid extraction (SFE)	The extraction is facilitated by means of reaching a supercritical state which occurs when the temperature and pressure of the fluid are raised above its critical point.	carbon dioxide co-solvent	fast, selective and solvent saving
Accelerated solvent extraction (ASE)	To accelerate extraction, it involves solvents under high temperature and pressure but without reaching the critical point.	methanol ethanol acetone	more suitable for the extraction of phenolics compounds than UAE

**Table 2:** A brief summary of current evidence of studies regarding the preventive and therapeutic effects of isoflavones and soyfoods.

Studies (Ref. No.)	Study Design	Contents		Main Preventive (P) and Therapeutic (T) Effects
**Hot flushes**				
[32] St Germain	randomized controlled trial (RCT)	soy	T	no difference
[33] Tice	RCT	isoflavone tablets	T	no difference
[35] Cancellieri	RCT	isoflavone from herbal supplement	T	isoflavones more effective than placebo
[34] Cheng	prospective study	isoflavones extracted from soya bean	T	isoflavones more effective than placebo
[16] Welty	RCT, crossover	soy nut	T	soy more effective than placebo
[36] Thomas	systematic review	natural vs. synthetic isoflavones	T	synthetic or combination isoflavones more effective than natural soy
[37] Washburn	randomized crossover trial	soy protein	T	soy protein more effective than placebo
[38] Khaodhiar	RCT	daidzein-rich isoflavone aglycones	T	daidzein-rich isoflavone aglycones more effective than placebo
[39] Cianci	observational prospective study	calcium, vitamin D3, inulin, soy isoflavones	T	soy supplement + inulin effective
[40] Carmignani	RCT	soy vs. hormone replacement therapy (HRT)	T	HRT more effective than soy; both are superior to placebo
[41] Bolanos-Dıaz	meta-analysis	soy extracts vs. HRT	T	HRT more effective than soy extracts; both are superior to placebo
[42] Amato	multicenter RCT	aglycone hypocotyl soy isoflavone	T	no difference
[43] Daily	systematic review, meta-analysis	soy isoflavone and equol	T	equol or isoflavone in equol-producers more effective than placebo
[28] Newton	observational study	equol-producer status	T	soy in equol-producers more effective than non-producers
[19] Lambert	RCT	red clover extracts	T	red clover extracts more effective than placebo
**Hormone-related osteoporosis**				
[45] Ma	meta-analysis	isoflavone	T	increase spinal bone mineral density (BMD)
[42] Amato	multicenter RCT	aglycone hypocotyl soy isoflavone	T	slow BMD loss
[44] Lambert	systematic review and meta-analysis	isoflavone aglycone	T	preserve BMD
**Urogenital tract**				
[48] Reed	RCT	black cohosh or dietary soy	T	no effect on vaginal cytology
[46] Waetjen	prospective cohort study	dietary intake of isoflavones	T	no effect on stress or urge incontinence
[49] Vitale	prospective, randomized, placebo-controlled study	isoflavones, calcium, vitamin D, inulin	T	May improve sexual function
[50] Ribeiro	RCT	isoflavone	T	failed to yield an estrogenic effect on the urogenital tract and to relieve the vulvovaginal symptoms
**Metabolic syndrome**				
[51] Stuenkel	randomized clinical trial	isoflavone supplements	T	loss of weight and fat mass, but interpretation difficult
[52] Mueller	in vitro study	PPARγ binding and transactivational activity	T	red clover extracts may be used to treat metabolic syndrome
**Cardiovascular disease**				
[53] van der Schouw	prospective study	food phytoestrogens	P	low dose phytoestrogen not protective
[54] Nestel	randomized crossover trial	purified soybean extract	T	may improve systemic arterial compliance
[34] Cheng	prospective study	isoflavones extracted from soya bean	T	no difference in lipoprotein lipids
[56] Wong	RCT	soy hypocotyl isoflavones	T	no effect on nitric oxide metabolism or blood pressure
[57] Suparto	animal study	soy protein	T	HRT + soy harmful, soy or HRT not beneficial
[58] Sathyapalan	double-blind randomized study	soy protein +/− soy isoflavone	T	soy protein with isoflavones improved cardiovascular markers compared to soy protein alone
[59] Ma	3 prospective cohort studies	isoflavones, tofu and soymilk	P	higher intake of isoflavones and tofu was associated with a moderately lower risk of developing coronary heart disease
**Cognitive function and neuromuscular systems**				
[61] Clement	systematic review	isoflavones and soy	T	may improve cognition
[60] Greendale	cohort study	dietary phytoestrogens	T	better processing speed but worse verbal memory
[62] Miyake	cross-sectional study	soy products and isoflavones	T	independent inverse relationships between intake of soy products and isoflavones and depressive symptoms during pregnancy
[63] Tabata	animal study	isoflavone aglycone	T	significantly modulated muscle atrophy after denervation in mice, probably due to the decrease in apoptosis-dependent signaling
**Cancer risk**				
[67] Hirose	case-control study	soy products as part of daily intake	P	lower risk of breast cancer in premenopausal women
[68] Alipour	case-control study	soy extracts		soy extracts may cause benign changes in breast
[71] Kang	cohort study	dietary intake of soy isoflavones	P	lower recurrence of estrogen- and progesterone-receptor positive breast cancers receiving anastrazole therapy after surgery
[76] Shin	case-control study	dietary soyfood and isoflavone intake	P	reduced risk for overall colorectal cancer
[70] Zhao	meta-analysis	soy foods	P	a high dietary intake of soy foods may reduce breast cancer risk
[72] Budhathoki	prospective study	soy food and isoflavone		were not associated with the risk of endometrial cancer.
[73] Quaas	double-blind RCT	isoflavone soy protein		no effect on the rates of endometrial hyperplasia and cancer
[74] Ollberding	prospective study	isoflavone, daidzein and genistein intake	P	are associated with a reduced risk of endometrial cancer
[75] Wada	population-based prospective study	total soy and isoflavones	P	are associated with a decreased risk of bladder cancer

## References

[1] United Nations Conference on Trade and Development: Soy Beans. https://unctad.org/en/PublicationsLibrary/INFOCOMM_cp10_SoyaBeans_en.pdf.

[2] Asbridge D.D., Erickson D.R. (1995). Chapter 1-soybeans vs. other vegetable oils as a source of edible oil products. Practical Handbook of Soybean Processing and Utilization.

[3] Johnson L.A., White P.J., Galloway R. (2008). Soybeans: Chemistry, Production, Processing, and Utilization.

[4] Coward L., Smith M., Kirk M., Barnes S. (1998). Chemical modification of isoflavones in soyfoods during cooking and processing. Am. J. Clin. Nutr..

[5] Kurosu M., El-Shemy H. (2011). Chapter 10-Biologically Active Molecules from Soybeans. Soybean and Health.

[6] Ligor M., Ratiu I.A., Kiełbasa A., Al-Suod H., Buszewski B. (2018). Extraction approaches used for the determination of biologically active compounds (cyclitols, polyphenols and saponins) isolated from plant material. Electrophoresis.

[7] Zhang Y.C., Schwartz S.J. (2003). Analysis of isoflavones in soy foods. Curr. Protoc. Food Anal. Chem..

[8] Krebs E.E., Ensrud K.E., MacDonald R., Wilt T.J. (2004). Phytoestrogens for treatment of menopausal symptoms: A systematic review. Obstet. Gynecol..

[9] Croden J., Ross S., Yuksel N., Sydora B.C. (2015). A survey of the availability in Canadian pharmacy chains of over-the-counter natural health products for menopause symptoms. BMC Complement. Altern. Med..

[10] Russell L., Hicks G.S., Low A.K., Shepherd J.M., Brown C.A. (2002). Phytoestrogens: A viable option?. Am. J. Med. Sci..

[11] Guo P.P., Li P., Zhang X.H., Liu N., Wang J., Chen D.D., Sun W.J., Zhang W. (2019). Complementary and alternative medicine for natural and treatment-induced vasomotor symptoms: An overview of systematic reviews and meta-analyses. Complement. Ther. Clin. Pract..

[12] Hill D.A., Crider M., Hill S.R. (2016). Hormone therapy and other treatments for symptoms of menopause. Am. Fam. Physician.

[13] Chen L.R., Ko N.Y., Chen K.H. (2019). Medical Treatment for osteoporosis: From molecular to clinical opinions. Int. J. Mol. Sci..

[14] National Osteoporosis Foundation (2010). Clinician’s Guide to Prevention and Treatment of Osteoporosis.

[15] De Franciscis P., Colacurci N., Riemma G., Conte A., Pittana E., Guida M., Schiattarella A. (2019). A nutraceutical approach to menopausal complaints. Medicina.

[16] Welty F.K., Lee K.S., Lew N.S., Nasca M., Zhou J.R. (2007). The association between soy nut consumption and decreased menopausal symptoms. J. Womens Health (Larchmt).

[17] Reed S.D., Lampe J.W., Qu C., Gundersen G., Fuller S., Copeland W.K., Newton K.M. (2013). Self-reported menopausal symptoms in a racially diverse population and soy food consumption. Maturitas.

[18] Ewies A.A.A. (2002). Phytoestrogens in the management of the menopause: Up-to-date. Obstet. Gynecol. Surv..

[19] Lambert M.N.T., Thorup A.C., Hansen E.S.S., Jeppesen P.B. (2017). Combined Red Clover isoflavones and probiotics potently reduce menopausal vasomotor symptoms. PLoS ONE.

[20] Britt K.L., Simpson E.R., Findlay J.K. (2005). Effects of phytoestrogens on the ovarian and pituitary phenotypes of estrogen-deficient female aromatase knockout mice. Menopause.

[21] Levis S., Strickman-Stein N., Doerge D.R., Krischer J. (2010). Design and baseline characteristics of the soy phytoestrogens as replacement estrogen (SPARE) study—A clinical trial of the effects of soy isoflavones in menopausal women. Contemp. Clin. Trials.

[22] Ashlock L., Rodibaugh R., Hettiarachchy N., Proctor A. (2000). Chapter 18: Processing and utilization. Arkansas Soybean Production Handbook.

[23] Zhang J., Ge Y., Han F., Li B., Yan S., Sun J., Wang L. (2014). Isoflavone content of soybean cultivars from maturity group 0 to VI grown in northern and southern China. J. Am. Oil. Chem. Soc..

[24] North American Menopause Society (2000). The role of isoflavones in menopausal health: Consensus opinion of The North American Menopause Society. Menopause.

[25] Tsourounis C. (2001). Clinical effects of phytoestrogens. Clin. Obstet. Gynecol..

[26] North American Menopause Society (2011). The role of soy isoflavones in menopausal health: Report of The North American Menopause Society/Wulf H. Utian Translational Science Symposium in Chicago, IL (October 2010). Menopause.

[27] Jackson R.L., Greiwe J.S., Desai P.B., Schwen R.J. (2011). Single-dose and steady-state pharmacokinetic studies of S-equol, a potent nonhormonal, estrogen receptor β-agonist being developed for the treatment of menopausal symptoms. Menopause.

[28] Newton K.M., Reed S.D., Uchiyama S., Qu C., Ueno T., Iwashita S., Gunderson G., Fuller S., Lampe J.W. (2015). A cross-sectional study of equol producer status and self-reported vasomotor symptoms. Menopause.

[29] Setchell K.D.R. (2017). The history and basic science development of soy isoflavones. Menopause.

[30] Guadamuro L., Dohrmann A.B., Tebbe C.C., Mayo B., Delgado S. (2017). Bacterial communities and metabolic activity of faecal cultures from equol producer and non-producer menopausal women under treatment with soy isoflavones. BMC Microbiol..

[31] Ishiwata N., Melby M.K., Mizuno S., Watanabe S. (2009). New equol supplement for relieving menopausal symptoms: Randomized, placebo-controlled trial of Japanese women. Menopause.

[32] St Germain A., Peterson C.T., Robinson J.G., Alekel D.L. (2001). Isoflavone-rich or isoflavone-poor soy protein does not reduce menopausal symptoms during 24 weeks of treatment. Menopause.

[33] Tice J.A., Ettinger B., Ensrud K., Wallace R., Blackwell T., Cummings S.R. (2003). Phytoestrogen supplements for the treatment of hot flashes: The Isoflavone Clover Extract (ICE) Study: A randomized controlled trial. JAMA.

[34] Cheng G., Wilczek B., Warner M., Gustafsson J.-A., Landgren B.M. (2007). Isoflavone treatment for acute menopausal symptoms. Menopause.

[35] Cancellieri F., De Leo V., Genazzani A.D., Nappi C., Parenti G.L., Polatti F., Ragni N., Savoca S., Teglio L., Finelli F. (2007). Efficacy on menopausal neurovegetative symptoms and some plasma lipids blood levels of an herbal product containing isoflavones and other plant extracts. Maturitas.

[36] Thomas A., Ismail R., Taylor-Swanson L., Cray L., Schnall J.G., Mitchell E.S., Woods N.F. (2014). Effects of isoflavones and amino acid therapies for hot flashes and co-occurring symptoms during the menopausal transition and early post menopause: A systematic review. Maturitas.

[37] Washburn S., Burke G.L., Morgan T., Anthony M. (1999). Effect of soy protein supplementation on serum lipoproteins, blood pressure, and menopausal symptoms in perimenopausal women. Menopause.

[38] Khaodhiar L., Ricciotti H.A., Li L., Pan W., Schickel M., Zhou J., Blackburn G.L. (2008). Daidzein-rich isoflavone aglycones are potentially effective in reducing hot flashes in menopausal women. Menopause.

[39] Cianci A., Colacurci N., Paoletti A.M., Perino A., Cicinelli E., Maffei S., Di Martino M., Daguati R., Stomati M., Pilloni M. (2015). Soy isoflavones, inulin, calcium, and vitamin D3 in post-menopausal hot flushes: An observational study. Clin. Exp. Obstet. Gynecol..

[40] Carmignani L.O., Pedro A.O., Costa-Paiva L.H., Pinto-Neto A.M. (2010). The effect of dietary soy supplementation compared to estrogen and placebo on menopausal symptoms: A randomized controlled trial. Maturitas.

[41] Bolaños-Díaz R., Zavala-Gonzales J.-C., Mezones-Holguín E., Francia-Romero J. (2011). Soy extracts versus hormone therapy for reduction of menopausal hot flushes: Indirect comparison. Menopause.

[42] Amato P., Young R.L., Steinberg F.M., Murray M.J., Lewis R.D., Cramer M.A., Barnes S., Ellis K.J., Shypailo R.J., Fraley J.K. (2013). Effect of soy isoflavone supplementation on menopausal quality of life. Menopause.

[43] Daily J.W., Ko B.S., Ryuk J., Liu M., Zhang W., Park S. (2019). Equol decreases hot flashes in postmenopausal women: A systematic review and meta-analysis of randomized clinical trials. J. Med. Food.

[44] Lambert M.N.T., Hu L.M., Jeppesen P.B. (2017). A systematic review and meta-analysis of the effects of isoflavone formulations against estrogen-deficient bone resorption in peri- and postmenopausal women. Am. J. Clin. Nutr..

[45] Ma D.-F., Qin L.-Q., Wang P.-Y., Katoh R. (2008). Soy isoflavone intake increases bone mineral density in the spine of menopausal women: Meta-analysis of randomized controlled trials. Clin. Nutr..

[46] Waetjen L.E., Leung K., Crawford S.L., Huang M.-H., Gold E.B., Greendale G.A. (2013). Study of women’s health across the nation relationship between dietary phytoestrogens and development of urinary incontinence in midlife women. Menopause.

[47] Burton J.L., Wells M. (2002). The effect of phytoestrogens on the female genital tract. J. Clin. Pathol..

[48] Reed S.D., Newton K.M., LaCroix A.Z., Grothaus L.C., Grieco V.S., Ehrlich K. (2008). Vaginal, endometrial, and reproductive hormone findings: Randomized, placebo-controlled trial of black cohosh, multibotanical herbs, and dietary soy for vasomotor symptoms: The Herbal Alternatives for Menopause (HALT) Study. Menopause.

[49] Vitale S.G., Caruso S., Rapisarda A.M.C., Cianci S., Cianci A. (2018). Isoflavones, calcium, vitamin D and inulin improve quality of life, sexual function, body composition and metabolic parameters in menopausal women: Result from a prospective, randomized, placebo-controlled, parallel-group study. Prz. Menopauzalny.

[50] Ribeiro A.E., Monteiro N.E.S., Moraes A.V.G., Costa-Paiva L.H., Pedro A.O. (2018). Can the use of probiotics in association with isoflavone improve the symptoms of genitourinary syndrome of menopause? Results from a randomized controlled trial. Menopause.

[51] Stuenkel C.A. (2007). Isoflavones and cardiovascular risk in postmenopausal women: No free lunch. Menopause.

[52] Mueller M., Jungbauer A. (2008). Red clover extract: A putative source for simultaneous treatment of menopausal disorders and the metabolic syndrome. Menopause.

[53] Van der Schouw Y.T., Kreijkamp-Kaspers S., Peeters P.H.M., Keinan-Boker L., Rimm E.B., Grobbee D.E. (2005). Prospective study on usual dietary phytoestrogen intake and cardiovascular disease risk in Western women. Circulation.

[54] Nestel P.J., Yamashita T., Sasahara T., Pomeroy S., Dart A., Komesaroff P., Owen A., Abbey M. (1997). Soy isoflavones improve systemic arterial compliance but not plasma lipids in menopausal and perimenopausal women. Arterioscler. Thromb. Vasc. Biol..

[55] Brzezinski A., Danenberg H.D. (2008). Sex hormones, soy, and myocardial injury. Menopause.

[56] Wong W.W., Taylor A.A., Smith E.O., Barnes S., Hachey D.L. (2012). Effect of soy isoflavone supplementation on nitric oxide metabolism and blood pressure in menopausal women. Am. J. Clin. Nutr..

[57] Suparto I.H., Williams J.K., Fox J.L., Yusuf J.T.L., Sajuthi D. (2008). Effects of hormone therapy and dietary soy on myocardial ischemia/reperfusion injury in ovariectomized atherosclerotic monkeys. Menopause.

[58] Sathyapalan T., Aye M., Rigby A.S., Thatcher N.J., Dargham S.R., Kilpatrick E.S., Atkin S.L. (2018). Soy isoflavones improve cardiovascular disease risk markers in women during the early menopause. Nutr. Metab. Cardiovasc. Dis..

[59] Ma L., Liu G., Ding M., Zong G., Hu F.B., Willett W.C., Rimm E.B., Manson J.E., Sun Q. (2020). Isoflavone intake and the risk of coronary heart disease in US men and women: Results from 3 prospective cohort studies. Circulation.

[60] Greendale G.A., Huang M.-H., Leung K., Crawford S.L., Gold E.B., Wight R., Waetjen E., Karlamangla A.S. (2012). Dietary phytoestrogen intakes and cognitive function during the menopausal transition: Results from the study of women’s health across the nation phytoestrogen Study. Menopause.

[61] Clement Y.N., Onakpoya I., Hung S.K., Ernst E. (2011). Effects of herbal and dietary supplements on cognition in menopause: A systematic review. Maturitas.

[62] Miyake Y., Tanaka K., Okubo H., Sasaki S., Furukawa S., Arakawa M. (2018). Soy isoflavone intake and prevalence of depressive symptoms during pregnancy in Japan: Baseline data from the Kyushu Okinawa Maternal and Child Health Study. Eur. J. Nutr..

[63] Tabata S., Aizawa M., Kinoshita M., Ito Y., Kawamura Y., Takebe M., Pan W., Sakuma K. (2019). The influence of isoflavone for denervation-induced muscle atrophy. Eur. J. Nutr..

[64] Rachoń D., Menche A., Vortherms T., Seidlová-Wuttke D., Wuttke W. (2008). Effects of dietary equol administration on the mammary gland in ovariectomized Sprague-Dawley rats. Menopause.

[65] Michikawa T., Yamazaki S., Ono M., Kuroda T., Nakayama S.F., Suda E., Isobe T., Iwai-Shimada M., Kobayashi Y., Yonemoto J. (2019). Isoflavone intake in early pregnancy and hypospadias in the Japan environment and children’s study. Urology.

[66] Reiter E., Beck V., Medjakovic S., Mueller M., Jungbauer A. (2009). Comparison of hormonal activity of isoflavone-containing supplements used to treat menopausal complaints. Menopause.

[67] Hirose K., Imaeda N., Tokudome Y., Goto C., Wakai K., Matsuo K., Ito H., Toyama T., Iwata H., Tokudome S. (2005). Soybean products and reduction of breast cancer risk: A case–control study in Japan. Br. J. Cancer.

[68] Alipour S., Afshar S., Moini A., Dastjerdi M., Saberi A., Bayani L., Eslami B., Hosseini L. (2012). Clinical and ultrasonographic changes of the breast after use of soy isoflavones. APJCP.

[69] Shike M., Doane A.S., Russo L., Cabal R., Reis-Filho J.S., Gerald W., Cody H., Khanin R., Bromberg J., Norton L. (2014). The effects of soy supplementation on gene expression in breast cancer: A randomized placebo-controlled study. J. Natl. Cancer Inst..

[70] Zhao T.T., Jin F., Li J.G., Xu Y.Y., Dong H.T., Liu Q., Xing P., Zhu G.L., Xu H., Miao Z.F. (2019). Dietary isoflavones or isoflavone-rich food intake and breast cancer risk: A meta-analysis of prospective cohort studies. Clin. Nutr..

[71] Kang X., Zhang Q., Wang S., Huang X., Jin S. (2010). Effect of soy isoflavones on breast cancer recurrence and death for patients receiving adjuvant endocrine therapy. CMAJ.

[72] Budhathoki S., Iwasaki M., Sawada N., Yamaji T., Shimazu T., Sasazuki S., Inoue M., Tsugane S., JPHC Study Group (2015). Soy food and isoflavone intake and endometrial cancer risk: The Japan Public Health Center-based prospective study. BJOG.

[73] Quaas A.M., Kono N., Mack W.J., Hodis H.N., Felix J.C., Paulson R.J., Shoupe D. (2013). Effect of isoflavone soy protein supplementation on endometrial thickness, hyperplasia, and endometrial cancer risk in postmenopausal women: A randomized controlled trial. Menopause.

[74] Ollberding N.J., Lim U., Wilkens L.R., Setiawan V.W., Shvetsov Y.B., Henderson B.E., Kolonel L.N., Goodman M.T. (2012). Legume, soy, tofu, and isoflavone intake and endometrial cancer risk in postmenopausal women in the multiethnic cohort study. J. Natl. Cancer Inst..

[75] Wada K., Tsuji M., Tamura T., Konishi K., Goto Y., Mizuta F., Koda S., Uji T., Hori A., Tanabashi S. (2018). Soy isoflavone intake and bladder cancer risk in Japan: From the Takayama study. Cancer Epidemiol. Biomark. Prev..

[76] Shin A., Lee J., Lee J., Park M.S., Park J.W., Park S.C., Oh J.H., Kim J. (2015). Isoflavone and soyfood intake and colorectal cancer risk: A case-control study in Korea. PLoS ONE.

[77] Eden J.A. (2012). Phytoestrogens for menopausal symptoms: A review. Maturitas.

[78] Bolaños R., Del Castillo A., Francia J. (2010). Soy isoflavones versus placebo in the treatment of climacteric vasomotor symptoms: Systematic review and meta-analysis. Menopause.

[79] Nelson H.D., Vesco K.K., Haney E., Fu R., Nedrow A., Miller J., Nicolaidis C., Walker M., Humphrey L. (2006). Nonhormonal therapies for menopausal hot flashes: Systematic review and meta-analysis. JAMA.

[80] Taku K., Melby M.K., Kronenberg F., Kurzer M.S., Messina M. (2012). Extracted or synthesized soybean isoflavones reduce menopausal hot flash frequency and severity: Systematic review and meta-analysis of randomized controlled trials. Menopause.

[81] Li L., Lv Y., Xu L., Zheng Q. (2015). Quantitative efficacy of soy isoflavones on menopausal hot flashes. Br. J. Clin. Pharmacol..

[82] Wuttke W., Jarry H., Seidlová-Wuttke D. (2007). Isoflavones—Safe food additives or dangerous drugs?. Ageing Res. Rev..

[83] Messina M. (2014). Soybean isoflavones warrant greater consideration as a treatment for the alleviation of menopausal hot flashes. Womens Health (Lond.).

[84] Utian W.H., Jones M., Setchell K.D.R. (2015). S-equol: A potential nonhormonal agent for menopause-related symptom relief. J. Womens Health (Larchmt).

[85] Crawford S.L., Jackson E.A., Churchill L., Lampe J.W., Leung K., Ockene J.K. (2013). The impact of dose, frequency of administration, and equol production on efficacy of isoflavones for menopausal hot flashes: A pilot randomized trial. Menopause.

[86] Su Wei Poh M., Voon Chen Yong P., Viseswaran N., Chia Y.Y. (2015). Estrogenicity of glabridin in Ishikawa cells. PLoS ONE.

